# Mitochondrial oxidative stress drives tumor progression and metastasis: should we use antioxidants as a key component of cancer treatment and prevention?

**DOI:** 10.1186/1741-7015-9-62

**Published:** 2011-05-23

**Authors:** Federica Sotgia, Ubaldo E Martinez-Outschoorn, Michael P Lisanti

**Affiliations:** 1 The Jefferson Stem Cell Biology and Regenerative Medicine Center, Philadelphia, PA, USA; 2 Departments of Stem Cell Biology & Regenerative Medicine, and Cancer Biology, Thomas Jefferson University, Philadelphia, PA, USA; 3 Manchester Breast Centre and Breakthrough Breast Cancer Research Unit, Paterson Institute for Cancer Research, Manchester, UK; 4 Department of Medical Oncology, Kimmel Cancer Center, Thomas Jefferson University, Philadelphia, PA, USA

## Abstract

The functional role of oxidative stress in cancer pathogenesis has long been a hotly debated topic. A study published this month in *BMC Cancer *by Goh *et al.*, directly addresses this issue by using a molecular genetic approach, via an established mouse animal model of human breast cancer. More specifically, alleviation of mitochondrial oxidative stress, via transgenic over-expression of catalase (an anti-oxidant enzyme) targeted to mitochondria, was sufficient to lower tumor grade (from high-to-low) and to dramatically reduce metastatic tumor burden by >12-fold. Here, we discuss these new findings and place them in the context of several other recent studies showing that oxidative stress directly contributes to tumor progression and metastasis. These results have important clinical and translational significance, as most current chemo-therapeutic agents and radiation therapy increase oxidative stress, and, therefore, could help drive tumor recurrence and metastasis. Similarly, chemo- and radiation-therapy both increase the risk for developing a secondary malignancy, such as leukemia and/or lymphoma. To effectively reduce mitochondrial oxidative stress, medical oncologists should now re-consider the use of powerful anti-oxidants as a key component of patient therapy and cancer prevention.

Please see related research article: http://www.biomedcentral.com/1471-2407/11/191

## Introduction

Mitochondrial oxidative stress has long been implicated in normal aging, and a host of human diseases, including cancer and neurodegenerative disorders, such as Alzheimer's disease. In support of this idea, vegetarians, who consume a diet rich in anti-oxidants, have reduced rates of cancer incidence, have longer life expectancies, and suffer less from dementia [1-3].

Similarly, breast cancer patients taking anti-oxidants showed reduced rates of recurrence, as well as less risk of mortality [4]. In fact, N-acetyl-cysteine (NAC), a powerful anti-oxidant, has anti-tumor properties, and has been recommended for melanoma chemo-prevention [5]. Finally, metformin therapy, a powerful anti-oxidant which reduces reactive oxygen species (ROS) production from mitochondrial complex I, has been associated with a lower risk of various epithelial cancers, in more than 11 studies [6,7].

A simple PubMed search reveals that nearly 9,000 articles have been published linking oxidative stress with cancer pathogenesis. Thus, it is surprising that anti-oxidants are not routinely used as a component of cancer therapy and prevention.

## Genetic reduction of mitochondrial oxidative stress reduces tumor grade and inhibits metastasis

This month in *BMC Cancer*, Goh and colleagues [8] use an established mouse model of breast cancer tumor formation and metastasis (MMTV-PyMT) to explore the role of mitochondrial oxidative stress in cancer pathogenesis. To reduce mitochondrial oxidative stress, they targeted a powerful anti-oxidant protein (catalase; which inactivates hydrogen peroxide) to mitochondria. This was achieved by modifying catalase with the addition of an N-terminal mitochondrial targeting signal and the deletion of a C-terminal peroxisome targeting sequence. Transgenic mice harboring mito-catalase have been previously shown to have an extended lifespan, consistent with the idea that mitochondrial oxidative stress directly contributes to normal aging [9].

Remarkably, MMTV-PyMT mice expressing mito-catalase showed a significant reduction in tumor grade (from high-grade to low-grade), and a dramatic reduction in lung metastatic tumor burden (>12-fold). Thus, it appears that genetic reductions in mitochondrial oxidative stress prevent i) normal aging, as well as ii) tumor progression, and iii) metastasis. Regardless of the exact mechanism, their results suggest that we should be treating cancer patients with powerful anti-oxidants, as a form of chemotherapy (either alone or following other therapies).

Previous studies evaluating the use of anti-oxidants in breast cancer patients have shown mixed results [4,10,11]. However, this may be because some studies are population-based without standardized treatments and/or only certain subtypes of breast cancer are sensitive to anti-oxidants. For example, breast cancers with a loss of stromal caveolin-1 (Cav-1) generate higher levels of reactive oxygen species (ROS) [12-14], as compared to breast cancers expressing high levels of stromal Cav-1. Loss of stromal Cav-1 is predictive of recurrence, metastasis, and poor clinical outcome, and as such is a new biomarker for breast and prostate cancer [15,16].

## How does mitochondrial oxidative stress drive tumor growth and metastasis?

What are the possible mechanism(s) by which mitochondrial oxidative stress contributes to tumor initiation and progression? Since the transgenic over-expression of "mitochondrial" catalase in these experiments is targeted to the whole body, it remains unknown whether the findings of Goh *et al. *[8] are related to reductions in oxidative stress in epithelial cancer cells, in the surrounding stromal cells, or in both cellular compartments.

**Figure 1:**
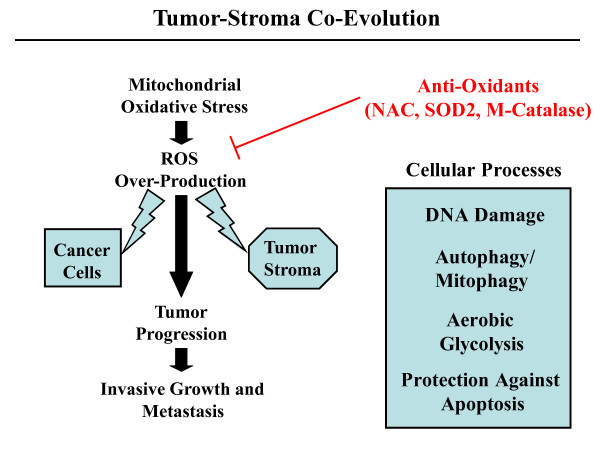
**Tumor evolution is fueled by mitochondrial oxidative stress**. The experiments by Goh *et al. *directly show that blocking mitochondrial ROS inhibits metastasis, indicating that mitochondrial oxidative stress promotes tumor progression and metastasis. The observed effects most likely involve the effects of ROS on both cancer cells and their surrounding tumor stroma. Cellular processes activated by ROS include DNA damage, autophagy/mitophagy, and aerobic glycolysis. Complementary studies have shown that ROS-induced activation of autophagy and aerobic glycolysis in cancer associated fibroblasts provide recycled nutrients (pyruvate, lactate, ketones, and glutamine, among others) for anabolic cancer cell growth, and protects these cancer cells against apoptosis. Importantly, anti-oxidants will prevent the oxidative stress, reducing tumor progression and metastasis. NAC, N-acetyl-cysteine; SOD2, mitochondrial superoxide dismutase; M-catalase, mitochondrially targeted catalase.

One possibility is that mitochondrial oxidative stress in epithelial cancer cells leads to ROS production and ensuing DNA damage, resulting in an increased mutation rate and tumor evolution, via the positive selection of tumor cell mutations that confer a growth advantage (Figure 1). In support of this notion, there is substantial evidence that another anti-oxidant enzyme, namely mitochondrial SOD2 (which de-activates super-oxide), behaves as a potent tumor suppressor protein [17-19].

Another possibility is that mitochondrial oxidative stress in tumor stromal cells, such as cancer associated fibroblasts, may have genetic and metabolic consequences that promote tumor growth (Figure 1).

In fact, MMTV-PyMT mice require an activated tumor stroma for the development of metastasis. Cross-talk between macrophages and epithelial cells via CSF-1 and EGF ligands is needed for progression [20]. Similarly, activation of their fibroblastic stroma by deletion of the Cav-1 gene (thereby increasing oxidative stress [12]) significantly promotes lung metastasis (>4-fold) in MMTV-PyMT mice [21,22].

More recent studies have shown that cancer cells induce oxidative stress in adjacent stromal fibroblasts, thereby conferring the cancer associated fibroblast phenotype [12,23]. Oxidative stress (due to ROS over-production) in cancer associated fibroblasts then leads to genetic instability in adjacent cancer cells (DNA damage and aneuploidy) via a "bystander effect", driving tumor-stroma co-evolution [12]. ROS over-production in cancer associated fibroblasts also drives the onset of autophagy and mitophagy in these cells, resulting in aerobic glycolysis, with lactate and ketone production (the "Reverse Warburg Effect") [14]. Energy-rich metabolites (lactate, pyruvate, ketones, and glutamine) are then transferred to "hungry" cancer cells, promoting mitochondrial biogenesis and anabolic growth in these tumor cells [24]. This event, in turn, promotes tumor growth and protects these cancer cells against apoptosis [12-14]. This new model of tumorigenesis has been termed "The Autophagic Tumor Stroma Model of Cancer Metabolism" [25,26]. The *in vivo *relevance of this model for breast and prostate cancer has been confirmed using the new biomarker Cav-1 [15,16,27]. When stromal Cav-1 is lost in cancer associated fibroblasts (due to the onset of oxidative stress, hypoxia, and/or autophagy) [12-14,28,29], this is highly predictive of tumor recurrence, metastasis, and drug-resistance [15]. For example, triple negative breast cancer patients with high stromal Cav-1 have a 12-year survival rate of >75% [30]. In contrast, triple negative breast cancer patients with a loss of stromal Cav-1 have a five-year survival rate of < 10% [30].

The association between loss of stromal Cav-1 and oxidative stress in the tumor stroma has also been confirmed by transcriptional profiling and bears a striking resemblance to the gene expression profiles of Alzheimer's disease (which is also directly linked to oxidative stress) [31,32]. In fact, when the gene profile of Alzheimer's disease brain was intersected with the transcriptional profiles from the tumor stroma of patients with breast cancer, this was sufficient to identify which breast cancer patients would undergo metastasis [32]. Thus, oxidative stress is common to both of these biological processes (cancer and neuro-degeneration), and may underlie their pathogenesis [31,32].

In order to further test the validity of this model and its dependence on mitochondrial oxidative stress, Capozza, Lisanti, and colleagues over-expressed mitochondrial SOD2 in cancer associated fibroblasts [33]. As predicted, over-expression of mitochondrial SOD2 in cancer associated fibroblasts was indeed sufficient to inhibit tumor growth by nearly two-fold [33]. These results indicate that mitochondrial SOD2 also behaves as a tumor suppressor in the stromal microenvironment [33]. Importantly, cytoplasmic SOD1 was ineffective in this stromal context, supporting a specific role for mitochondrial oxidative stress.

Similarly, oxidative stress is known to be sufficient to convert normal fibroblasts to myo-fibroblasts or cancer associated fibroblasts, via activation of two key transcription factors, namely HIF1-alpha and NFkB [12,14,34-36]. Thus, one way to genetically pheno-copy the effects of oxidative stress is to over-express activated forms of HIF1-alpha or NFkB [34]. As such, fibroblasts expressing activated HIF1-alpha or NFkB are sufficient to promote tumor growth, up to three-fold [34]. Furthermore, activation of HIF-alpha or NFkB in fibroblasts drives a loss of stromal Cav-1 via lysosomal degradation, and activates the autophagic program resulting in mitophagy, a shift towards aerobic glycolysis, and lactate production [34]. Fibroblasts harboring activated HIF1-alpha or NFkB, then provide lactate and other recycled nutrients to feed cancer cells [34].

Additional lines of evidence also support the idea that oxidative stress in the tumor stroma plays a key role in cancer pathogenesis and tumor spreading. For example, there are numerous papers directly showing that local or systemic administration of purified anti-oxidant proteins (that is, catalase or SOD) is sufficient to block tumor recurrence and distant metastasis in multiple cancer models [37-39].

## Conclusions

In order to maximize treatment benefits, we will need to develop new biomarkers (like stromal Cav-1) to predict which cancer patients will respond best to anti-oxidant therapy. Unfortunately, most medical oncologists now recommend against taking anti-oxidants during cancer therapy, as it "may reduce the effectiveness of chemotherapies", which are largely based on increasing oxidative stress [4]. However, in direct contradiction of this recommendation, a recent breast cancer study directly shows that anti-oxidant therapy significantly reduces breast cancer recurrence and mortality [4].

Thus, reductions in mitochondrial oxidative stress in both cancer cells and their surrounding tumor stroma may be beneficial for preventing tumor progression and metastasis. Ultimately, this "new concept" could radically change how we treat cancer patients, and stimulate new anti-oxidant strategies for cancer prevention.

## Abbreviations

HIF1: hypoxia-inducible factor 1; MMTV: mouse mammary tumor virus; PyMT: polyoma middle T antigen; ROS: reactive oxygen species; SOD2: superoxide dismutase 2, mitochondrial enzyme

## Competing interests

The authors declare that they have no competing interests.

## Authors' contributions

FS, UEM-O and MPL all contributed equally to the writing and editing of this commentary. All authors read and approved the final manuscript.

## Pre-publication history

The pre-publication history for this paper can be accessed here:

http://www.biomedcentral.com/1741-7015/9/62/prepub
